# Dietary Fat and Polyunsaturated Fatty Acid Intakes during Childhood Are Prospectively Associated with Puberty Timing Independent of Dietary Protein

**DOI:** 10.3390/nu14020275

**Published:** 2022-01-10

**Authors:** Yujie Xu, Jingyuan Xiong, Wanke Gao, Xiaoyu Wang, Shufang Shan, Li Zhao, Guo Cheng

**Affiliations:** 1 Department of Nutrition and Food Safety, West China School of Public Health and West China Fourth Hospital, Sichuan University, Chengdu 610041, China; jayee1217@126.com (Y.X.); gaowanke_gwk@126.com (W.G.); 2 Healthy Food Evaluation Research Center, West China School of Public Health and West China Fourth Hospital, Sichuan University, Chengdu 610041, China; jzx0004@tigermail.auburn.edu; 3 Laboratory of Molecular Translational Medicine, Center for Translational Medicine, Key Laboratory of Birth Defects and Related Diseases of Women and Children, Ministry of Education, Department of Pediatrics, West China Second University Hospital, Sichuan University, Chengdu 610041, China; wangxiaoyu12168@163.com (X.W.); shan103163@163.com (S.S.); 4 Department of Health Policy and Management, West China School of Public Health and West China Fourth Hospital, Sichuan University, Chengdu 610041, China; zhaoli@scu.edu.cn

**Keywords:** dietary fat, polyunsaturated fatty acid, monounsaturated fatty acid, puberty timing

## Abstract

Dietary fat and fat quality have been inconsistently associated with puberty timing. The aim of this study was to investigate the prospective associations of dietary fat, saturated fatty acid (SFA), polyunsaturated fatty acid (PUFA), and monounsaturated fatty acid (MUFA) with puberty timing. Using longitudinal data from China Health and Nutrition Survey (CHNS) and Southwest China Childhood Nutrition and Growth (SCCNG) Study, we analyzed dietary data, anthropometric measurements, and potential confounders. Dietary intakes were assessed by 3-day 24-h recalls. Age at Tanner stage 2 for breast/genital development (B2/G2) and age at menarche/voice break (M/VB) were used as puberty development markers. Cox proportional hazard regression models were used to estimate the relevance of dietary intake of total fat, SFA, PUFA, and MUFA on puberty timing. Among 3425 girls and 2495 boys, children with higher intakes of total fat and PUFA were more likely to reach their B2/G2 or M/VB at an earlier age. Associations were not attenuated on additional adjustment for childhood dietary protein intake. However, higher intakes of SFA or MUFA were not independently associated with puberty development. A higher intake of dietary fat and PUFA in prepuberty was associated with earlier puberty timing, which was independent of dietary protein intake.

## 1. Introduction

Puberty represents the transition stage from childhood to adulthood [1]. There has been a global secular trend towards earlier puberty timing in the past decades [2,3,4]. Such a trend is concerning because individuals with an earlier puberty onset are at a higher risk of diabetes, cardiovascular diseases, hormone-related cancers, and all cause-mortality in later life [5,6]. Identifying modifiable factors of puberty timing is thus critical to public health ramifications, and nutrition is the most relevant to determine the timing of puberty among these modifiable influences [7].

Observational studies have demonstrated that dietary intakes of food groups or nutrients during childhood are associated with puberty timing [8,9,10]. Although dietary fat has been reported to alter sex hormones in humans [11,12], prospective associations regarding dietary fat and the onset of puberty are limited and conflicting. A prospective study has found an inverse association between childhood total fat intake and age at puberty onset [13], whereas other studies have reported no association [14,15], or positive association [16]. From a public health standpoint, it is more meaningful to investigate the possible effect of fat quality, i.e., saturated fatty acid (SFA), polyunsaturated fatty acid (PUFA), and monounsaturated fatty acid (MUFA), other than total fat [7], but the relationship between different types of fatty acids and puberty timing varies: some studies found that higher consumption of PUFA was associated with early puberty onset [8,17], while others reported a lack of an association [16,18]; no association between SFA intake with puberty timing was observed [8,16,18]; inverse [15], positive [17] or null association [16] were reported between MUFA intake and age at puberty onset. Notably, most of these studies were limited mainly to girls and examined solely a single self-report puberty marker [8,13,14,15,16,17,18], usually age at menarche, which highlights the need to consider other puberty timing traits reflecting different sex hormone pathways [19]. The measurement of different pubertal markers covering the range from earlier (Tanner stage 2 for breast development in girls or genital development in boys (B2/G2)) to later stages (menarche in girls or voice break in boys (M/VB)) of pubertal development might provide more comprehensive pubertal data. Furthermore, those previous studies were conducted in Caucasian from Western countries [8,13,14,15,16,17,18], including the British [8], Germany [13], America [14,17], Canada [16], and the Netherlands [18]; little is known about the impact of dietary fat and specific types of fatty acids on puberty timing in Chinese children. Given the secular trend of earlier puberty has been observed in both Chinese boys and girls [20,21], investigation on the determinants of puberty timing in this population is warranted.

Currently, the obesity epidemic has received large attention, and body composition in childhood has turned out to be associated with puberty timing [22]. In recent years, China has witnessed a secular increase in childhood obesity [23]. Owing to the two trends that coincide in China, the impact of dietary fat on puberty timing beyond body composition needs to be investigated. In addition, fat-rich foods, such as red meat, beans, and peanuts, are also the predominant nutritional source of dietary protein, and whose effect on the timing of sexual maturation has been largely confirmed [10]. However, few studies handled appropriately the independent association of dietary fat on the timing of puberty. Hence, it is important to examine whether a potential effect of dietary fat or fatty acids on puberty onset is mediated by childhood dietary protein intake.

Using longitudinal data from the China Health and Nutrition Survey (CHNS) and the Southwest China Childhood Nutrition and Growth (SCCNG) Study, we thus aimed to investigate the prospective associations between dietary fat and fatty acids intake and earlier puberty markers (B2/G2) or later puberty markers (M/VB) in Chinese boys and girls. Moreover, we also examined whether these associations were independent of pre-pubertal dietary protein intake.

## 2. Materials and Methods

### 2.1. Study Sample

We used nationally representative data from the CHNS (1997–2015) and the SCCNG studies.

The CHNS is an ongoing, large-scale, and longitudinal study conducted in China. Fifteen provinces were selected, and a multi-stage random cluster sampling method was performed in the ten waves between 1989 to 2015. A detailed protocol of the cohort has been published elsewhere [24]. Since data on B2 in girls or G2 and VB in boys were not available in the CHNS, we only included girls with menarche information in the present study. Based on girls with available data of the occurrence of menarche, our analysis considered 2405 girls aged 6–13 years that were recruited between 1997 to 2015. Of these participants, 1433 girls with complete dietary assessment were included for baseline. Then, 173 girls with incomplete information on household income, anthropometry, or other potential confounders, and 19 girls with implausible energy intakes (<600 kcal/day and >4000 kcal/day) [25] were excluded. Finally, 1240 CHNS girls were eligible in the present analysis.

The SCCNG study was started in March 2013 and covered three provinces (Sichuan province, Guizhou province, and Chongqing province) in southwest China, aiming to investigate the development and nutritional status of Chinese children. The study procedure was described in detail elsewhere [26]. The yearly recruitment from 2013 onward enrolled children aged 6–8 years who were cooperative and voluntary. At the first examination, we collected information on sociodemographic issues, dietary intake and eating behaviors, physical activity and sedentary behaviors, anthropometry, and pubertal development. From then on, the participants were followed up for assessment on nutritional status and growth at regular intervals until the age of 15. Detailed information on the assessments of anthropometry and puberty status were obtained every year, while data of dietary intake and physical activity were collected biennially. The study was approved by the Ethics Committee of the Sichuan University, and all the participants gave their written confirmed consent before enrollment. Between January 2013 and December 2019, 5439 children aged 6–8 years had baseline dietary information and completed at least 2 follow-up assessments. Since we were interested in the prospective relevance of dietary fat intake on puberty timing, 389 children who had already reached B2/G2 at baseline were excluded. Then, 268 participants with implausible energy intakes (<600 kcal/day and >4000 kcal/day) [25] and 102 participants with incomplete information on potential confounders were further excluded. A final sample of 4680 children (2185 girls and 2495 boys) was included in this analysis.

**Figure 1:**
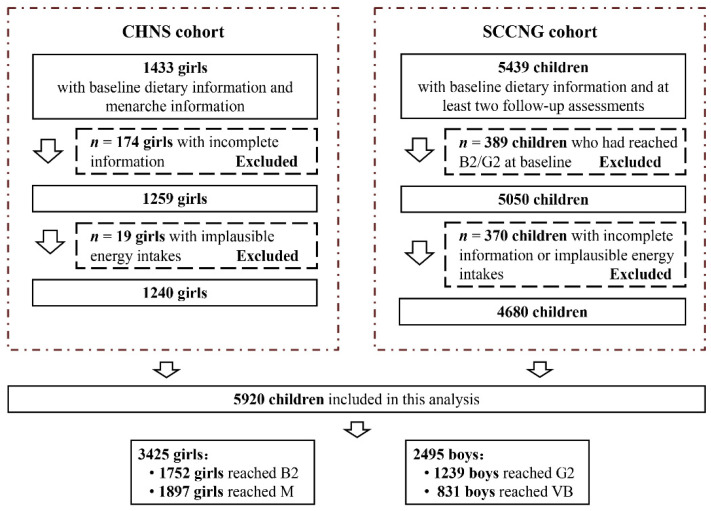
Flow chart for the study sample in our study. CHNS, China Health and Nutrition Survey; SCCNG, Southwest China Childhood Nutrition and Growth; B2, Tanner stage 2 for breast development; G2, Tanner stage for genital development; M, menarche; VB, voice break.

Hence, we included 5920 children (1240 girls from CHNS, 4680 children from SCCNG) in the present study; 1752 girls reached B2, 1897 girls (732 girls from CHNS, 1165 girls from SCCNG) experienced menarche, and 1239 boys reached G2, 831 boys experienced voice break. The flow chart of the sample selection was shown in Figure 1.

### 2.2. Nutrition Assessment

Nutritional data of children in CHNS and SCCNG were collected by trained investigators via 3-day 24-h dietary recalls. In CHNS, when children were 12 years or older, they were asked to recall their consumption of all foods and beverages. For children younger than 12 years, dietary intake data from school were provided by themselves while the information on food consumption at home was provided by their parents. In SCCNG, children aged 9 years or older were asked to recall their consumption of all foods and beverages. For children younger than 9 years, parents provided the information on food consumption at home, while children provided the dietary intake information from school themselves.

Details on recipes and brands of all food items reported were inquired. Food models, standard tableware including bowls, plates, and glasses, and picture aids were provided to enhance the accuracy of portion size estimates [27]. In the SCCNG study, a designed photo book that contains photos of snacks and beverages and pictures of the commonly used commercial packaging to improve the accuracy of diet recall was also given to children.

Nutrition data of children that were collected via 3-day 24-h dietary recalls recorded their consumption of foods and beverages, including categories and quantities. Then we transformed this original information to energy and specific nutrient intake by calculating the nutrient content in every food item based on the Chinese food composition (FCT). In CHNS, FCT 1991 was used in the 1997 and 2000 dietary survey; FCT 2002, FCT 2004, and 2009 (combined) were used in the 2004, 2006, 2009, and 2011 dietary survey, respectively. While in SCCNG, dietary intake data were converted into energy and nutrient intake data using the continuously updated in-house nutrient database based on NCCW software (version 11.0, 2014), which reflects the FCT.

### 2.3. Assessment of Puberty Onset

In the CHNS study, girls aged 8 years or older were asked whether their menarche had already occurred and the detail on the month and year of the first menstrual period were recorded in each survey. If there were different reported menarche ages for one girl, we only included the first reported menarche age in the panel data for analysis to reduce potential recall bias. In the SCCNG study, pubertal maturation for breast (girls) and pubic hair (girls and boys) stages were assessed at each examination by investigators according to the standardized criteria of Tanner stages. Testicular volume was assessed by comparative palpation with the Prader orchidometer. If the testicular volumes of the two testes were not equal, the volume of the larger one was recorded. Testicular volume less than 1 mL was recorded as 1 mL. In addition, during the annual physical examination, children were asked whether menarche (girls) or voice break (boys) had already occurred, and the respective month and year were recorded.

For this analysis, age at B2 in girls and the initiation of G2 in boys as well as age at menarche in girls and age at voice break in boys were considered.

### 2.4. Anthropometry

In CHNS and SCCNG study, anthropometric measurements of the participants were performed at each visit by trained research assistants according to standard procedures, with the subjects dressed lightly and barefoot. Height and weight were measured to the nearest 0.1 cm and 0.1 kg, respectively. Body mass index (BMI) was calculated as weight divided by the square of height (kg/m^2^). All anthropometric measurements were performed twice, and the averages were calculated for each child.

For this analysis, sex- and age-independent BMI SDS and age-specific BMI Z-score were calculated for each children using the equation by Cole et al. [28] based on a Chinese reference population [29]. Overweight was defined according to the International Obesity Task Force (IOTF) BMI cut-offs for children, which correspond to an adult BMI of 25 kg/m^2^ [28].

### 2.5. Covariates

In both CHNS and SCNNG study, detailed information about pregnancy and infancy (i.e., children’s birth weight, exclusive breastfeeding duration, the timing of complementary feeding), and family characteristics (i.e., place of residence, household income, family size, smoking in the household, parental age, parental occupation, and parental education levels) were collected using structured questionnaires.

### 2.6. Statistical Analysis

SAS^®^ procedures (version 9.4, SAS Inc., Cary, NC, USA) were used for all data analyses. All analyses were performed with a significance level at *p* < 0.05.

Dietary fat intakes were expressed as sex- and age-specific residuals from the regression of dietary fat intakes on energy intake. To examine the potential associations of dietary fat intakes with puberty timing, their distribution was grouped into tertiles (T1–T3), with the lowest tertile serving as the reference group. T1 indicates the lowest tertile who have a lowest intake of dietary fat, while T3 indicates the highest tertile with those having the highest dietary fat consumption.

Kolmogorov-Smirnov and Shapiro-Wilk tests were conducted to test the normality of the data. The continuous variables were normally distributed and presented as means (SD). Differences in anthropometric data, socio-demographic data, and nutritional intake between genders were tested using ANOVA test for normally distributed continuous variables, Kruskal-Wallis test for not normally distributed continuous variables, Chi-square test was used for categorical variables, followed by Student-Newman-Keuls tests or Dunn’s post hoc tests. Since different pubertal markers were used in boys and girls, statistical models, as well as descriptive tables, were stratified by sex.

To investigate the prospective relevance of dietary fat intakes at baseline with age at B2/G2 or M/VB, Cox proportional hazard regression models were used. Censoring occurred at the age of reaching B2/G2 and M/VB or age at last follow-up if the puberty events had not been reported. In the basic models, the tertiles of dietary fat intakes at baseline were the principle independent fixed effects. Cox regression models were adjusted for birth year, age at baseline, body composition (Z-scores of BMI, overweight (Y/N)), family income, parental educational level, mother’s age at menarche (only from SCCNG population), and total energy intake at baseline and dietary protein intake (residuals) at baseline. Each potential confounder was initially considered separately and included if it was associated with both the dietary index and indicators of puberty timing and substantially altered the estimate by more than 10%. Thus, birth year, family income level, energy intake at baseline, and mother’s age at menarche (for B2, VB, and G2 model) were adjusted for in model 2. In the final model, Z-scores of BMI at baseline and dietary protein intake (residuals) at baseline were considered. Hazard ratios (HRs) and 95% confidence intervals (CIs) were estimated by comparing the second and third tertiles to the first tertile in these models.

## 3. Results

This study analyzed data on 5920 children (3425 girls and 2495 boys). There were no significant differences in age, or the BMI SDS at baseline among these 5920 children.

Table 1 summarized anthropometric, parental, and nutritional characteristics, and puberty timing in girls and boys. The mean age at baseline was 7.0 (0.8) years for girls from SCCNG and CHNS, and 7.1 (0.8) years for boys from SCCNG. Most puberty traits occurred earlier in girls than boys. Among girls from SCCNG and CHNS, 1748 (51.0%) had reached B2 at a mean (SD) age of 9.2 (1.4) years, and 1897 (55.4%) had experienced menarche at age of 12.6 (0.7) years. Among boys from SCCNG, 1233 (49.4%) had reached G2 aged 11.2 (1.1) years, and 829 (33.2%) had experienced voice break at a mean age of 13.7 (1.0) years. Compared to boys from SCCNG, girls from SCCNG and CHNS had a lower prevalence of overweight (10.1% of the girls and 11.9% of the boys) and were more likely to have a highly educated father and mother and grow up in a family with high income. As for childhood dietary energy and macronutrients intakes, boys from SCCNG consumed more total energy, dietary fat, and more dietary SFA, MUFA, and PUFA compared with girls from SCCNG and CHNS.

The associations of childhood dietary fat intakes at baseline with early and late markers of puberty development were presented in Table 2. According to the Cox proportional hazard regression model adjusted for birth year, family income level, energy intake, BMI Z-score, and dietary protein intake at baseline, girls with a higher intake of dietary fat were more likely to reach their B2 (adjusted HR = 1.13, 95% CI, 1.07 to 1.21, *p* for trend = 0.03) or experience their menarche (adjusted HR = 1.17, 95% CI, 1.11 to 1.23, *p* for trend = 0.01) earlier than those with a lower intake of dietary fat. Similarly, boys who consumed more dietary fat experienced their G2 (adjusted HR = 1.09, 95% CI, 1.03 to 1.15, *p* for trend = 0.03) and voice break (adjusted HR = 1.12, 95% CI, 1.07 to 1.16, *p* for trend = 0.03) at an earlier age compared with boys with a low dietary fat intake. In both genders, these associations were independent of dietary protein intake in childhood.

Furthermore, we examined the effect of the three dietary fatty acids, i.e., SFA, MUFA, and PUFA, on the puberty timing in girls and boys. Table 3 revealed significant associations of childhood PUFA intake with early and late puberty markers in both genders: girls with a higher PUFA intake had approximately 11% higher HR to reach their B2 (adjusted HR = 1.11, 95% CI, 1.05 to 1.17, *p* for trend = 0.02) or 13% higher HR to experience their menarche (adjusted HR = 1.13, 95% CI, 1.08 to 1.20, *p* for trend = 0.03) than girls with a lower PUFA intake. Similarly, compared to boys with a lower PUFA intake, boys who consumed more PUFA had approximately 8% higher HR to reach G2 (adjusted HR = 1.08, 95% CI, 1.03 to 1.13, *p* for trend = 0.03) or 10% higher HR to experience voice break (adjusted HR = 1.10, 95% CI, 1.06 to 1.15, *p* for trend = 0.02). However, no association of SFA intake with age at B2/G2 or M/VB was found in any model for both genders (Table S1). Moreover, the associations between MUFA intakes in childhood and pubertal development in our participants were presented in Table S2: higher MUFA intake in girls was associated with earlier B2 (adjusted HR = 1.10, 95% CI, 1.04 to 1.18, *p* for trend = 0.04) and earlier menarche (adjusted HR = 1.11, 95% CI, 1.06 to 1.17, *p* for trend = 0.05) in a model adjusted for parental and childhood characteristics. Nevertheless, further adjustment for dietary protein intakes eliminated these associations, indicating that the effect of MUFA, unlike PUFA, on puberty timing was not independent of dietary protein intake. In contrast, no association of MUFA with puberty onset was seen in boys in any model.

## 4. Discussion

We observed that higher childhood habitual total fat and PUFA intakes were associated with subsequent earlier timing of puberty, which was independent of pre-puberty dietary protein intake. However, MUFA or SFA was not associated with any puberty markers.

Our findings with total fat and earlier puberty are comparable with previous cohort studies conducted in Germany [13] and America [31]. Data from 261 German girls aged 8–15 years showed that girls with higher consumption of total fat were associated with accelerated menarche [13]. Based on data from 64 Caucasian American boys, childhood fat and animal protein intakes were found to be predictive of earlier age at peak height velocity [31]. This may lie in the fact that fat intake has the potential to accelerate the maturation of the hypothalamic–pituitary–gonadal axis and result in differences in hormone concentrations [32]. However, other prospective cohort studies that conducted in girls from the USA [17,33,34,35], Canada [15,16,36], Greece [36], and New Zealand [18] or boys from Australia [37] and the UK [38] reported inconsistent conclusions with our analysis. The reasons behind the discrepancies are likely to be multifactorial. The differences in genetic background, sample size, and dietary assessment may partly lead to inconsistent results. Our study is the first report of the Asian population with 3425 girls and 2495 boys, while the participants of others ranged from 67 to 2299. Larger sample size was achieved in our study and thus might have greater statistical power to reveal diet–puberty associations than most previous studies. Moreover, main food sources of dietary fat, such as red meat and milk, are also the predominant resources of dietary protein and have been demonstrated to be inversely associated with puberty timing [9,39]. However, the influence of dietary protein on the association of dietary fat with puberty timing has not been excluded appropriately in these previous analyses [9,15,16,17,18,33,34,35,37,38,39,40]. Since dietary protein intake is associated with puberty timing [10], we adjusted for childhood dietary protein intake in the Cox model. In this way, we could figure out the independent impact of dietary fat on puberty development, and we believe that our findings are helpful to build public health initiatives of dietary fat intake.

We also extended previous evidence of associations between dietary fat quality and puberty timing [8,15,16,17,18,40]. Our findings with PUFA and SFA are broadly consistent with conclusions of an earlier meta-analysis in 2020 which reported an inverse association of PUFA and null association of SFA with menarche in girls [10] based on cohorts in the USA [17] and Canada [15,16,36]. Another cohort of 3872 UK girls [41] and a cohort including 63 Caucasian girls [18] also demonstrated the inverse association of PUFA intake and null association of SFA intake with age at B2, respectively. Nevertheless, most of the previous reports [15,16,17,18,40] on associations between PUFA or SFA and puberty timing did not adjust for child body composition, which was associated with puberty development [22]. In our analysis, we have overcome this common limitation and presented more convincing conclusions after adjusting for parental characteristics, child body composition, and other potential confounders. Randomized trials in boys and girls also confirmed the null association between SFA and puberty timing [42,43]. In addition, we described associations of higher PUFA intake and higher odds of earlier puberty onset in boys, which was inconsistent with the only existing data from Avon Longitudinal Study of Parents and Children (ALSPC) cohort boys from the UK [41]. The reasons for the difference may be as follows. On one hand, the average timing of G2 (mean (SD), 8.7 (1.6) years) reported in the ALSPC cohort was unusually earlier compared to that reported in non-Hispanic white (10.1 (2.1) years) and Hispanic (10.0 (1.8) years) boys [44], which indicated that the subsample in ALSPC cohort was not a good representation of the total Caucasian boys, and the null association of PUFA based on these data should be considered with caution; while boys in our study entered G2 at a mean age of 11.2 (1.1) and close to that (10.55 (95% confidential intervals, 10.27 to 10.79)) reported in 18,807 urban Chinese boys [20], thus our investigation on diet-puberty association had a good representative sample. On the other hand, the mean PUFA intake (11.0 (1.3) g/day vs. 17.7 (5.2) g/day) and age at G2 (8.7 (1.6) years vs. 11.2 (1.1) years) of participants, nutritional assessment instrument (3-day food diaries vs. 3-day 24-h recalls), and statistical method (multivariable linear regression model vs. Cox proportional hazard regression model) were quite different between ALSPC cohort and our study, which could partly interpret this discrepancy as well. For MUFA, we found that there is no association with puberty onset in girls and boys, which has been reported in existing cohorts [16,41].

Furthermore, except Cheng TS et al. [41], most of the previous studies [8,15,16,17,18] have not ruled out the effect of dietary protein intake on the association between SFA, PUFA, or MUFA and puberty timing. We considered dietary protein intake at baseline in the multivariable model using the residual method and figured out the inverse association of PUFA, which was independent of dietary protein intake. Similar to our results, Cheng TS et al. reported the independent effect of PUFA using an isocaloric substitution model [41]. However, the exact mechanisms elucidating the association between PUFA intakes and puberty timing remain to be determined. A higher intake of PUFA may affect steroidogenic machinery and mammary gland development to accelerate growth and reproductive progress [45]. In vitro, PUFA modulates adrenal steroidogenesis and acts on the production of adrenal androgen, which eventually stimulates gonadotropin-releasing hormone neurons that are required for puberty onset [46]. In vivo, an increased ratio of n-6 to n-3 PUFA (5:1) modulated the reproductive function in female zebrafish [47]. Our findings failed to draw a conclusion on specific PUFA, and the optimal ratio of PUFA merited further investigation. We also found that there were no independent association of SFA with the puberty timing, but interestingly, the inverse association between MUFA intake and puberty in girls was changed in the model additionally adjusting for dietary protein, i.e., this association was mediated by dietary protein intake. Specific associations between SFA, MUFA, or PUFA and puberty timing suggest that there may be differences in the origin, metabolism, and biological activity of fatty acids [48]. Children usually get most of their dietary protein from animal foods, like red meat, milk, and eggs, which are also rich in SFA and MUFA, but relatively less in PUFA [49]. Similar data reported that children consumed most of their PUFA from fish, beans, and peanuts, but these foods have a relatively low concentration of SFA and MUFA [50]. This might partly explain why we observed an independent association for PUFA, but not for SFA or MUFA, and the previous association observed of MUFA and puberty timing may be driven by the dietary protein [15,17]. Meanwhile, MUFA can be sourced from the diet or endogenously synthesized from SFA [49], and Stearoyl-CoA desaturase (SCD) is the rate-limiting enzyme required for this synthesis. The expression of SCD1 gene has been reported to relate to the ratio of SFA and MUFA in vivo [51,52], and the polymorphisms of SCD1 gene are associated with SCD1 enzyme activity [53]. Thus, genetic variation of SCD1 gene among our participants would influence their endogenous MUFA levels. In aggregate, the interaction in SFA and MUFA metabolism may thus partly complicate the null association of SFA or MUFA in our study, although MUFA could theoretically stimulate mammary gland development. Hence, more studies that focus on the association of sex-specific individual fatty acids with pubertal development and the underlying pathway are needed.

Our study has several strengths. In contrast to most of the previous studies, we examined the puberty data from both girls and boys and provided a representable sample of Asian children. We also assessed multiple pubertal timing traits in both genders, showing a global view of puberty development. The prospective design, repeated follow-up for measurement on anthropometric, pubertal, and dietary data, as well as necessary adjustment for potential cofounders both in children and their parents were other strengths. Among these confounders, adjustment for dietary protein intake was the major strength in our analysis.

Some limitations should be acknowledged. In the CHNS, data on parental pubertal (mother’s age at menarche) characteristics were not available, so we could not adjust for genetic influences; meanwhile, only the year of menarche occurrence in girls was considered in this study, therefore potential bias could not be avoided. However, our models have included a larger number of major potential confounders both in children and in their parents than most previous studies [15,16,17,18,33,34,35,36,40], which could partly overcome this potential limitation.

In conclusion, our analysis suggested that children with higher intakes of dietary fat and PUFA in prepuberty would enter their puberty at an earlier age. This association was independent of childhood dietary protein intake.

## Figures and Tables

**Table 1:** Characteristics ^1^ of participants in our study.

	Girls from SCCNG (*n* = 2185)	Girls from SCCNG and CHNS (*n* = 3425)	Boys from SCCNG (*n* = 2495)
Age at baseline (years)	7.1 (0.7)	7.0 (0.8)	7.1 (0.8)
Age at Tanner stage B2 ^2^ (years, *n* = 1748)	9.1 (1.3)	9.2 (1.4)	−
Age at Tanner stage G2 ^2^ (years, *n* = 1233)	−	−	11.2 (1.1)
Age at menarche (years, *n* = 1894)	12.9 (0.8)	12.6 (0.7)	−
Age at voice break (years, *n* = 829)	−	−	13.7 (1.0)
BMI SDS at baseline (kg/m^2^)	0.1 (0.8)	−0.1 (0.7)	0.2 (0.8) ^#^
Overweight ^3^ (*n* (%))	251 (11.5)	345 (10.1)	299 (11.9) ^#^
High physical activity (*n* (%))	572 (26.2)	791 (23.1)	671 (26.9)
**Parental data at baseline**			
High family income ^4^ (*n* (%))	478 (21.9)	688 (20.1)	578 (23.2) ^#^
High paternal educational level ^5^ (*n* (%))	463 (21.2)	671 (19.6)	646 (25.9) ^# ※^
High maternal educational level ^5^ (*n* (%))	412 (18.9)	602 (17.6)	489 (19.6) ^#^
Mother’s age at menarche (years)	12.3 (1.1)	−	12.3 (1.0)
**Nutritional data ^6^**			
Total energy intake (kcal/day)	1762 (237)	1729 (262)	1928 (267) ^# ※^
Fat (g/day)	51.2 (16.9)	50.3 (14.6)	57.6 (15.8) ^#^
Fat (% of energy)	26.9 (7.5)	26.2 (7.6)	26.9 (7.6)
Saturated fatty acids (g/day)	14.6 (3.0)	13.2 (5.6)	16.9 (5.6) ^#^
Saturated fatty acids (% of energy)	7.5 (1.8)	6.9 (1.6)	7.9 (1.6) ^#^
Monounsaturated fatty acids (g/day)	18.7 (4.2)	17.4 (4.6)	19.4 (6.2) ^#^
Monounsaturated fatty acids (% of energy)	9.6 (2.7)	9.1 (2.8)	9.1 (4.2)
Polyunsaturated fatty acids (g/day)	14.1 (4.1)	12.8 (2.7)	17.7 (5.2) ^# ※^
Polyunsaturated fatty acids (% of energy)	7.2 (2.1)	6.7 (1.7)	8.3 (2.7) ^# ※^
Carbohydrate (% of energy)	59.3 (7.2)	60.5 (8.4)	58.9 (8.0)
Protein (% of energy)	13.8 (2.4)	13.3 (2.1)	14.2 (2.3)

^1^ Values are means (SD) or frequency; test for difference between the groups was performed, using ANOVA test for normal distributed continuous variables, Kruskal-Wallis test for not normally distributed continuous variables, followed by Student-Newman-Keuls tests or Dunn’s post hoc tests, and Chi-square test for categorical variables. *p* < 0.05 between girls from SCCNG and girls from SCCNG and CHNS, ^#^ *p* < 0.05 between girls from SCCNG and CHNS and boys from SCCNG, ^※^ *p* < 0.05 between boys from SCCNG and girls from SCCNG; ^2^ Tanner stage 2 for breast development; ^3^ definition according to the International Obesity Task Force (IOTF) [28]; ^4^ average annual income of family income in each survey year was inflated to values in 2015 by adjusting for consumer price index and then divided into five groups (yuan): low (≤5000), middle (5000–10,000), and high (>10,000) [30]; ^5^ school education at least 12 years; ^6^ mean values of dietary data at baseline using 3-day 24 h recall.

**Table 2:** Association ^1^ of dietary fat intake in childhood with puberty timing.

		Dietary Fat Intake at Baseline		
**Girls**	**T1 ^2^**	**T2 ^2^**	**T3 ^2^**	***p_trend_* ^3^**
Age at Tanner stage B2 (*n* = 2185)				
Unadjusted model:	1	1.12 (1.06, 1.19)	1.18 (1.13, 1.24)	0.04
Model 2 ^4^:	1	1.13 (1.05, 1.18)	1.17 (1.11, 1.22)	0.03
Final model ^5^:	1	1.11 (1.03, 1.19)	1.13 (1.07, 1.21)	0.03
Age at menarche (*n* = 3425)				
Unadjusted model:	1	1.15 (1.09, 1.20)	1.20 (1.15, 1.26)	0.03
Model 2 ^6^:	1	1.17 (1.12, 1.22)	1.22 (1.17, 1.27)	0.02
Final model ^5^:	1	1.13 (1.06, 1.19)	1.17 (1.11, 1.23)	0.01
**Boys**	**T1 ^7^**	**T2 ^7^**	**T3 ^7^**	***p_trend_* ^3^**
Age at Tanner stage G2 (*n* = 2495)				
Unadjusted model:	1	1.09 (1.04, 1.13)	1.12 (1.08, 1.17)	0.04
Model 2 ^6^:	1	1.11 (1.05, 1.17)	1.15 (1.10, 1.21)	0.04
Final model ^5^:	1	1.07 (1.03, 1.12)	1.09 (1.03, 1.15)	0.03
Age at voice break (*n* = 2495)				
Unadjusted model:	1	1.11 (1.06, 1.18)	1.14 (1.09, 1.19)	0.04
Model 2 ^6^:	1	1.12 (1.08, 1.17)	1.16 (1.11, 1.21)	0.03
Final model ^5^:	1	1.08 (1.02, 1.13)	1.12 (1.07, 1.16)	0.03

^1^ Values are models adjusted hazard ratios (95% CI), HR = hazard ratio; ^2^ values are min-max in tertiles in girls for age at Tanner stage B2: T1 (12.7–39.2), T2 (39.6–64.7), and T3 (64.9–81.7); values are min-max in tertiles in girls for age at menarche: T1 (11.6–37.7), T2 (38.1–62.3), and T3 (62.5–76.3); ^3^ *p* for trend across tertiles were performed by including dietary fat intake at baseline as continuous variables; ^4^ adjusted for birth year, family income level and energy intake at baseline; ^5^ additionally adjusted for BMI Z-scores at baseline and dietary protein intake (residual) at baseline; ^6^ adjusted for birth year, family income level, energy intake at baseline and mother’s age at menarche; ^7^ values are min-max in tertiles in boys: T1 (15.8–47.2), T2 (47.3–69.5), and T3 (69.7–93.6).

**Table 3:** Association ^1^ of dietary polyunsaturated fatty acid (PUFA) in childhood with puberty timing.

	Dietary PUFA at Baseline			
**Girls**	**T1 ^2^**	**T2 ^2^**	**T3 ^2^**	***p_trend_* ^3^**
Age at Tanner stage B2 (*n* = 2185)				
Unadjusted model:	1	1.08 (1.03, 1.12)	1.13 (1.08, 1.19)	0.04
Model 2 ^4^:	1	1.09 (1.04, 1.13)	1.14 (1.09, 1.20)	0.03
Final model ^5^:	1	1.07 (1.02, 1.11)	1.11 (1.05, 1.17)	0.02
Age at menarche (*n* = 3425)				
Unadjusted model:	1	1.11 (1.07, 1.16)	1.18 (1.12, 1.22)	0.04
Model 2 ^6^:	1	1.13 (1.08, 1.19)	1.17 (1.12, 1.21)	0.03
Final model ^5^:	1	1.09 (1.06, 1.13)	1.13 (1.08, 1.20)	0.03
**Boys**	**T1 ^7^**	**T2 ^7^**	**T3 ^7^**	***p_trend_* ^3^**
Age at Tanner stage G2 (*n* = 2495)				
Unadjusted model:	1	1.08 (1.03, 1.13)	1.11 (1.05, 1.18)	0.04
Model 2 ^6^:	1	1.09 (1.03, 1.14)	1.12 (1.05, 1.20)	0.03
Final model ^5^:	1	1.06 (1.02, 1.11)	1.08 (1.03, 1.13)	0.03
Age at voice break (*n* = 2495)				
Unadjusted model:	1	1.09 (1.04, 1.13)	1.12 (1.06, 1.18)	0.03
Model 2 ^6^:	1	1.11 (1.06, 1.17)	1.13 (1.08, 1.19)	0.03
Final model ^5^:	1	1.07 (1.03, 1.12)	1.10 (1.06, 1.15)	0.02

^1^ Values are models adjusted hazard ratios (95% CI), HR = hazard ratio; ^2^ values are min-max in tertiles in girls for age at Tanner stage B2: T1 (4.3–8.2), T2 (8.3–15.6), and T3 (15.7–20.1); values are min-max in tertiles in girls for age at menarche: T1 (4.1–7.5), T2 (7.6–13.9), and T3 (14.0–18.6); ^3^ *p* for trend across tertiles were performed by including dietary fat intake at baseline as continuous variables; ^4^ adjusted for birth year, family income level and energy intake at baseline; ^5^ additionally adjusted for Z-scores of BMI at baseline and dietary protein intake (residual) at baseline; ^6^ adjusted for birth year, family income level, energy intake at baseline and mother’s age at menarche; ^7^ values are min-max in tertiles in boys: T1 (3.9–13.1), T2 (13.2–18.3), and T3 (18.4–21.6).

## Data Availability

Data from CHNS described in the manuscript will be made publicly and freely available without restriction at China Health and Nutrition Survey. Available online: https://www.cpc.unc.edu/projects/china/data/datasets/index.html (accessed on 1 January 2021). And data from SCCNG will be made available upon request pending approval by the corresponding author.

## Supplementary Materials

The following supporting information can be downloaded at: https://www.mdpi.com/article/10.3390/nu14020275/s1, Table S1: Association of dietary saturated fatty acid (SFA) in childhood with puberty timing, Table S2: Association of dietary monounsaturated fatty acid (MUFA) in childhood with puberty timing.

## References

[1] Patton G.C., Viner R. (2007). Pubertal transitions in health. Lancet.

[2] Euling S.Y., Herman-Giddens M.E., Lee P.A., Selevan S.G., Juul A., Sorensen T.I., Dunkel L., Himes J.H., Teilmann G., Swan S.H. (2008). Examination of US puberty-timing data from 1940 to 1994 for secular trends: Panel findings. Pediatrics.

[3] Ersoy B., Balkan C., Gunay T., Onag A., Egemen A. (2004). Effects of different socioeconomic conditions on menarche in Turkish female students. Early Hum. Dev..

[4] Prentice S., Fulford A.J., Jarjou L.M., Goldberg G.R., Prentice A. (2010). Evidence for a downward secular trend in age of menarche in a rural Gambian population. Ann. Hum. Biol..

[5] Day F.R., Elks C.E., Murray A., Ong K.K., Perry J.R. (2015). Puberty timing associated with diabetes, cardiovascular disease and also diverse health outcomes in men and women: The UK Biobank study. Sci. Rep..

[6] Chen X., Liu Y., Sun X., Yin Z., Li H., Liu X., Zhang D., Cheng C., Liu L., Liu F. (2018). Age at menarche and risk of all-cause and cardiovascular mortality: A systematic review and dose-response meta-analysis. Menopause.

[7] Villamor E., Jansen E.C. (2016). Nutritional Determinants of the Timing of Puberty. Annu. Rev. Public Health.

[8] Rogers I.S., Northstone K., Dunger D.B., Cooper A.R., Ness A.R., Emmett P.M. (2010). Diet throughout childhood and age at menarche in a contemporary cohort of British girls. Public Health Nutr..

[9] Jansen E.C., Marín C., Mora-Plazas M., Villamor E. (2015). Higher Childhood Red Meat Intake Frequency Is Associated with Earlier Age at Menarche. J. Nutr..

[10] Nguyen N.T.K., Fan H.-Y., Tsai M.-C., Tung T.-H., Huynh Q.T.V., Huang S.-Y., Chen Y.C. (2020). Nutrient Intake through Childhood and Early Menarche Onset in Girls: Systematic Review and Meta-Analysis. Nutrients.

[11] Hämäläinen E., Adlercreutz H., Puska P., Pietinen P. (1984). Diet and serum sex hormones in healthy men. J. Steroid Biochem..

[12] Wu A.H., Pike M.C., Stram D.O. (1999). Meta-analysis: Dietary fat intake, serum estrogen levels, and the risk of breast cancer. J. Natl. Cancer Inst..

[13] Merzenich H., Boeing H., Wahrendorf J. (1993). Dietary fat and sports activity as determinants for age at menarche. Am. J. Epidemiol..

[14] Meyer F., Moisan J., Marcoux D., Bouchard C. (1990). Dietary and physical determinants of menarche. Epidemiology.

[15] Moisan J., Meyer F., Gingras S. (1990). A nested case-control study of the correlates of early menarche. Am. J. Epidemiol..

[16] Koo M.M., Rohan T.E., Jain M., McLaughlin J.R., Corey P.N. (2002). A cohort study of dietary fibre intake and menarche. Public Health Nutr..

[17] Maclure M., Travis L.B., Willett W., MacMahon B. (1991). A prospective cohort study of nutrient intake and age at menarche. Am. J. Clin. Nutr..

[18] de Ridder C.M., Thijssen J.H., Van’t Veer P., van Duuren R., Bruning P.F., Zonderland M.L., Erich W.B. (1991). Dietary habits, sexual maturation, and plasma hormones in pubertal girls: A longitudinal study. Am. J. Clin. Nutr..

[19] Berenbaum S.A., Beltz A.M., Corley R. (2015). The importance of puberty for adolescent development: Conceptualization and measurement. Adv. Child Dev. Behav..

[20] Ma H.-M., Chen S.-K., Chen R.-M., Zhu C., Xiong F., Li T., Wang W., Liu G.-L., Luo X.-P., Liu L. (2011). Pubertal development timing in urban Chinese boys. Int. J. Androl..

[21] Ma H.-M., Du M.-L., Luo X.-P., Chen S.-K., Liu L., Chen R.-M., Zhu C., Xiong F., Li T., Wang W. (2009). Onset of Breast and Pubic Hair Development and Menses in Urban Chinese Girls. Pediatrics.

[22] Wagner I.V., Sabin M., Pfäffle R.W., Hiemisch A., Sergeyev E., Körner A., Kiess W. (2012). Effects of obesity on human sexual development. Nat. Rev. Endocrinol..

[23] Jia P., Xue H., Zhang J., Wang Y. (2017). Time Trend and Demographic and Geographic Disparities in Childhood Obesity Prevalence in China-Evidence from Twenty Years of Longitudinal Data. Int. J. Env. Res. Public Health.

[24] Popkin B.M., Du S., Zhai F., Zhang B. (2010). Cohort Profile: The China Health and Nutrition Survey—monitoring and understanding socio-economic and health change in China, 1989-2011. Int. J. Epidemiol..

[25] Mervish N.A., Teitelbaum S.L., Pajak A., Windham G.C., Pinney S.M., Kushi L.H., Biro F.M., Wolff M.S. (2017). Peripubertal dietary flavonol and lignan intake and age at menarche in a longitudinal cohort of girls. Pediatr Res..

[26] Yang M.Z., Xue H.M., Pan J., Libuda L., Muckelbauer R., Yang M., Quan L., Cheng G. (2018). High protein intake along with paternal part-time employment is associated with higher body fat mass among girls from South China. Eur. J. Nutr..

[27] Zhai F.Y., Du S.F., Wang Z.H., Zhang J.G., Du W.W., Popkin B.M. (2014). Dynamics of the Chinese diet and the role of urbanicity, 1991-2011. Obes. Rev..

[28] Cole T.J., Bellizzi M.C., Flegal K.M., Dietz W.H. (2000). Establishing a standard definition for child overweight and obesity worldwide: International survey. BMJ.

[29] Li H., Ji C.Y., Zong X.N., Zhang Y.Q. (2009). Body mass index growth curves for Chinese children and adolescents aged 0 to 18 years. Zhonghua Er Ke Za Zhi.

[30] National Bureau of Statistics of China (2020). China Statistical Yearbook.

[31] Alimujiang A., Colditz G.A., Gardner J.D., Park Y., Berkey C.S., Sutcliffe S. (2018). Childhood diet and growth in boys in relation to timing of puberty and adult height: The Longitudinal Studies of Child Health and Development. Cancer Causes Control..

[32] Lemarchand-Béraud T., Zufferey M.-M., Reymond M., Rey I. (1982). Maturation of the Hypothalamo-Pituitary-Ovarian Axis in Adolescent Girls. J. Clin. Endocrinol. Metab..

[33] Berkey C.S., Gardner J.D., Frazier A.L., Colditz G.A. (2000). Relation of childhood diet and body size to menarche and adolescent growth in girls. Am. J. Epidemiol..

[34] Koprowski C., Ross R.K., Mack W.J., Henderson B.E., Bernstein L. (1999). Diet, body size and menarche in a multiethnic cohort. Br. J. Cancer.

[35] Kissinger D.G., Sanchez A. (1987). The association of dietary factors with the age of menarche. Nutr. Res..

[36] Petridou E., Syrigou E., Toupadaki N., Zavitsanos X., Willett W., Trichopoulos D. (1996). Determinants of age at menarche as early life predictors of breast cancer risk. Int. J. Cancer.

[37] Cheng H.L., Raubenheimer D., Steinbeck K., Baur L., Garnett S. (2019). New insights into the association of mid-childhood macronutrient intake to pubertal development in adolescence using nutritional geometry. Br. J. Nutr..

[38] Cheng T.S., Sharp S.J., Brage S., Emmett P.M., Forouhi N.G., Ong K.K. (2021). Longitudinal associations between prepubertal childhood total energy and macronutrient intakes and subsequent puberty timing in UK boys and girls. Eur. J. Nutr..

[39] Ramezani Tehrani F., Moslehi N., Asghari G., Gholami R., Mirmiran P., Azizi F. (2013). Intake of dairy products, calcium, magnesium, and phosphorus in childhood and age at menarche in the Tehran Lipid and Glucose Study. PLoS ONE.

[40] Moisan J., Meyer F., Gingras S. (1990). Diet And Age At Menarche. Cancer Causes Control..

[41] Cheng T.S., Day F.R., Perry J.R.B., Luan J., Langenberg C., Forouhi N.G., Wareham N.J., Ong K.K. (2021). Prepubertal Dietary and Plasma Phospholipid Fatty Acids Related to Puberty Timing: Longitudinal Cohort and Mendelian Randomization Analyses. Nutrients.

[42] Sadov S., Virtanen H.E., Main K.M., Andersson A.M., Juul A., Jula A., Raitakari O.T., Pahkala K., Niinikoski H., Toppari J. (2019). Low-saturated-fat and low-cholesterol diet does not alter pubertal development and hormonal status in adolescents. Acta Paediatr..

[43] Niinikoski H., Lagstrom H., Jokinen E., Siltala M., Ronnemaa T., Viikari J., Raitakari O.T., Jula A., Marniemi J., Nanto-Salonen K. (2007). Impact of repeated dietary counseling between infancy and 14 years of age on dietary intakes and serum lipids and lipoproteins: The STRIP study. Circulation.

[44] Herman-Giddens M.E., Steffes J., Harris D., Slora E., Hussey M., Dowshen S.A., Wasserman R., Serwint J.R., Smitherman L., Reiter E.O. (2012). Secondary sexual characteristics in boys: Data from the Pediatric Research in Office Settings Network. Pediatrics.

[45] Anderson B.M., MacLennan M.B., Hillyer L.M., Ma D.W.L. (2014). Lifelong exposure to n-3 PUFA affects pubertal mammary gland development. Appl. Physiol. Nutr. Metab..

[46] Sampath H., Ntambi J.M. (2005). Polyunsaturated fatty acid regulation of genes of lipid metabolism. Annu. Rev. Nutr..

[47] Fowler L.A., Dennis-Cornelius L.N., Dawson J.A., Barry R.J., Davis J.L., Powell M.L., Yuan Y., Williams M.B., Makowsky R., D’Abramo L.R. (2020). Both Dietary Ratio of n-6 to n-3 Fatty Acids and Total Dietary Lipid Are Positively Associated with Adiposity and Reproductive Health in Zebrafish. Curr. Dev. Nutr..

[48] Tvrzicka E., Kremmyda L.S., Stankova B., Zak A. (2011). Fatty acids as biocompounds: Their role in human metabolism, health and disease—A review. Part 1: Classification, dietary sources and biological functions. Biomed. Pap. Med. Fac. Univ. Palacky Olomouc Czech Repub..

[49] Michas G., Micha R., Zampelas A. (2014). Dietary fats and cardiovascular disease: Putting together the pieces of a complicated puzzle. Atherosclerosis.

[50] Yaméogo C.W., Cichon B., Fabiansen C., Rytter M.J.H., Faurholt-Jepsen D., Stark K.D., Briend A., Shepherd S., Traoré A.S., Christensen V.B. (2017). Correlates of whole-blood polyunsaturated fatty acids among young children with moderate acute malnutrition. Nutr. J..

[51] Karahashi M., Ishii F., Yamazaki T., Imai K., Mitsumoto A., Kawashima Y., Kudo N. (2013). Up-regulation of stearoyl-CoA desaturase 1 increases liver MUFA content in obese Zucker but not Goto-Kakizaki rats. Lipids.

[52] Alim M.A., Fan Y.P., Wu X.P., Xie Y., Zhang Y., Zhang S.L., Sun D.X., Zhang Y., Zhang Q., Liu L. (2012). Genetic effects of stearoyl-coenzyme A desaturase (SCD) polymorphism on milk production traits in the Chinese dairy population. Mol. Biol. Rep..

[53] Martín-Núñez G.M., Cabrera-Mulero R., Rojo-Martínez G., Gómez-Zumaquero J.M., Chaves F.J., de Marco G., Soriguer F., Castaño L., Morcillo S. (2013). Polymorphisms in the SCD1 gene are associated with indices of stearoyl CoA desaturase activity and obesity: A prospective study. Mol. Nutr. Food Res..

