# Supplementary material for: Dietary Fat and Polyunsaturated Fatty Acid Intakes during Childhood Are Prospectively Associated with Puberty Timing Independent of Dietary Protein
Source: Nutrients. 2022 Jan 10;14(2):275. doi: 10.3390/nu14020275 (PMC8778261; doi:10.3390/nu14020275)
Supplement: Supplementary file 1 [file nutrients-14-00275-s001.zip › nutrients-1456179-supplementary.pdf]

Table S1. Association[1] of dietary saturated fatty acid (SFA) in childhood with puberty timing.

	Dietary SFA at baseline			p_{trend} [3]
	**T1**[2]	T2[2]	T3[2]	
**Girls**				
**Age at Tanner stage B2 (n=2185)**				
Unadjusted model:	1	1.04 (0.86, 1.16)	1.05 (0.97, 1.21)	0.07
Model 2[4]:	1	1.02 (0.82, 1.17)	1.03 (0.95, 1.23)	0.06
Final model[5]:	1	1.05 (0.98, 1.21)	1.07 (0.98, 1.19)	0.06
**Age at menarche (n=3425)**				
Unadjusted model:	1	0.95 (0.79, 1.15)	0.99 (0.87, 1.19)	0.06
Model 2[6]:	1	0.99 (0.80, 1.19)	1.04 (0.91, 1.22)	0.053
Final model[5]:	1	1.04 (0.92, 1.25)	1.08 (0.95, 1.25)	0.07
**Boys**	T1[7]	T2[7]	T3[7]	p_{trend} [3]
**Age at Tanner stage G2 (n=2495)**				
Unadjusted model:	1	0.92 (0.75, 1.13)	0.98 (0.85, 1.17)	0.08
Model 2[6]:	1	0.96 (0.78, 1.17)	1.02 (0.93, 1.21)	0.06
Final model[5]:	1	1.01 (0.91, 1.23)	1.05 (0.94, 1.26)	0.06
**Age at voice break (n=2495)**				
Unadjusted model:	1	0.94 (0.76, 1.17)	0.99 (0.89, 1.21)	0.08
Model 2[6]:	1	0.97 (0.77, 1.18)	1.03 (0.95, 1.27)	0.07
Final model[5]:	1	1.03 (0.93, 1.26)	1.06 (0.93, 1.23)	0.06

[1] Values are models adjusted hazard ratios (95% CI), HR= hazard ratio; [2] Values are min-max in tertiles in girls for age at Tanner stage B2: T1 (8.1-12.3), T2 (12.4-17.8), and T3 (17.9-23.1); values are min-max in tertiles in girls for age at menarche: T1 (7.8-10.9), T2 (11.0-15.2), and T3 (15.3-21.6); [3] P for trend across tertiles were performed by including dietary fat intake at baseline as continuous variables; [4] Adjusted for birth year, family income level and energy intake at baseline; [5] Additionally adjusted for Z-scores of BMI at baseline and dietary protein intake (residual) at baseline; [6] Adjusted for birth year, family income level, energy intake at baseline and mother's age at menarche; [7] Values are min-max in tertiles in boys: T1 (8.4-14.5), T2 (14.6-20.7), and T3 (20.8-27.2).

Table S2. Association[1] of dietary monounsaturated fatty acid (MUFA) in childhood with puberty timing.

	Dietary MUFA at baseline			
	**T1[2]**	T2[2]	T3[2]	p_{trend}[3]
**Girls**				
**Age at Tanner stage B2 (n=2185)**				
Unadjusted model:	1	1.07 (1.01 1.13)	1.09 (1.02, 1.17)	0.04
Model 2[4]:	1	1.06 (1.02, 1.11)	1.10 (1.04, 1.18)	0.04
Final model[5]:	1	1.04 (0.97, 1.13)	1.07 (0.98, 1.20)	0.06
**Age at menarche (n=3425)**				
Unadjusted model:	1	1.05 (0.99 1.16)	1.09 (1.01, 1.19)	0.06
Model 2[6]:	1	1.06 (1.01, 1.12)	1.11 (1.06, 1.17)	0.047
Final model[5]:	1	0.99 (0.87, 1.16)	1.06 (0.93, 1.21)	0.07
**Boys**	T1[7]	T2[7]	T3[7]	p_{trend}[3]
**Age at Tanner stage G2 (n=2495)**				
Unadjusted model:	1	0.93 (0.81, 1.10)	0.97 (0.89, 1.18)	0.07
Model 2[6]:	1	0.99 (0.89, 1.19)	1.03 (0.95, 1.16)	0.06
Final model[5]:	1	1.03 (0.96, 1.12)	1.04 (0.95, 1.21)	0.07
**Age at voice break (n=2495)**				
Unadjusted model:	1	1.02 (0.95, 1.18)	1.03 (0.96, 1.22)	0.07
Model 2[6]:	1	1.01 (0.92, 1.10)	1.05 (0.98, 1.25)	0.06
Final model[5]:	1	1.02 (0.95, 1.16)	1.04 (0.96, 1.23)	0.06

[1] Values are models adjusted hazard ratios (95% CI), HR= hazard ratio; [2] Values are min-max in tertiles in girls for age at Tanner stage B2: T1 (8.5-14.8), T2 (14.9-19.3), and T3 (19.4-25.2); values are min-max in tertiles in girls for age at menarche: T1 (8.1-13.9), T2 (14.0-18.9), and T3 (19.0-24.5); [3] P for trend across tertiles were performed by including dietary fat intake at baseline as continuous variables; [4] Adjusted for birth year, family income level and energy intake at baseline; [5] Additionally adjusted for Z-scores of BMI at baseline and dietary protein intake (residual) at baseline; [6] Adjusted for birth year, family income level, energy intake at baseline and mother's age at menarche; [7] Values are min-max in tertiles in boys: T1 (8.7-15.1), T2 (15.2-22.6), and T3 (22.7-31.6).
